# Hypokalemic Periodic Paralysis Associated With a Rare *CACNA1S* Variant (p.Leu1243Val): Expanding the Mutational Spectrum

**DOI:** 10.1155/crig/8824228

**Published:** 2026-01-15

**Authors:** Mark Abi Nader

**Affiliations:** ^1^ Department of Nephrology and Hypertension, Kidney Care Consultants, Memphis, Tennessee, USA

**Keywords:** *CACNA1S*, carbonic anhydrase inhibitors, genotype–phenotype correlation, hypokalemic periodic paralysis, hypophosphatemia, skeletal muscle channelopathy, variant reclassification

## Abstract

**Background:**

Hypokalemic periodic paralysis (HypoPP) is a rare skeletal muscle channelopathy, most often caused by mutations in *CACNA1S* or *SCN4A*. Most pathogenic *CACNA1S* mutations affect arginine residues in S4 voltage‐sensor domains, but other variants remain poorly understood.

**Case Presentation:**

I describe a 30‐year‐old Caucasian woman with recurrent paralytic episodes and hypokalemia (2.1–2.3 mmol/L), triggered by stress and carbohydrate‐rich meals. Genetic testing revealed heterozygosity for *CACNA1S* c.3727C  >  G (p.Leu1243Val), a variant of uncertain significance not previously associated with pathogenicity. Her phenotype was consistent with HypoPP. Treatment with spironolactone and acetazolamide reduced episode frequency, although the latter caused intolerable side effects, particularly tachypnea; she was later approved for dichlorphenamide. During one hospitalization, she also developed transient hypophosphatemia and hypokalemia, consistent with her HypoPP picture. Kidney function and imaging were normal. Family history revealed electrolyte disturbances in her grandfather.

**Conclusions:**

This case highlights a possible genotype–phenotype link involving *CACNA1S* p.Leu1243Val. Continued reporting of such cases is essential for variant reclassification and for improving recognition of metabolic shifts during HypoPP attacks.

## 1. Background

Hypokalemic periodic paralysis (HypoPP) is an uncommon autosomal‐dominant skeletal muscle channelopathy, with an estimated prevalence of 1 in 100,000 [1, 2]. It presents with episodic weakness linked to hypokalemia, often following carbohydrate‐rich meals, rest after exertion, or emotional stress.

About 60%–70% of cases are caused by *CACNA1S* mutations (HypoPP1) and 20% by *SCN4A* (HypoPP2) [3, 4]. Most *CACNA1S* variants involve arginine residues in the S4 voltage‐sensor segments, which generate abnormal gating‐pore (GP) currents that destabilize the resting potential [3, 4]. Classic examples include p.Arg528His and p.Arg1239His/Gly. However, additional loci continue to emerge, and clinical case reports remain crucial for determining pathogenicity [5–8].

## 2. Historical Background on GP Current Theory

Since 2007, the GP current mechanism has been recognized as the principal pathophysiologic basis of HypoPP [3]. Missense mutations affecting S4 arginine residues of *CACNA1S* (e.g., p.Arg528His, p.Arg1239His) generate aberrant “leak” currents during hyperpolarization, leading to paradoxical depolarization and muscle inexcitability [4]. Variants near these residues, such as p.Arg1242Gly, have been associated with atypical or normokalemic periodic paralysis (NormoPP) [16].

## 3. Treatment Overview

First‐line management includes trigger avoidance and oral potassium supplementation, followed by carbonic anhydrase inhibitors (CAIs) when episodes persist [9, 10]. Dichlorphenamide has proven efficacy in randomized controlled trials, while acetazolamide remains widely used [9, 11].

Carbohydrate‐triggered attacks may also involve transient hypophosphatemia, likely due to insulin‐mediated intracellular shifts of both potassium and phosphate [13–15].

Here, I present a young woman with HypoPP carrying *CACNA1S* c.3727C  >  G (p.Leu1243Val), currently classified as a variant of uncertain significance.

## 4. Case Presentation

The patient is a 30‐year‐old Caucasian woman with a history of migraines. She works in clinical research and is pursuing a PhD. In September 2024, she began experiencing recurrent episodes of diffuse muscle weakness, tingling, and cramps. Stress and high‐carbohydrate meals frequently triggered her symptoms.

During her first hospitalization, her serum potassium was 2.1–2.3 mmol/L. At that time, she was taking topiramate for migraine prophylaxis, which was discontinued. Despite cessation of topiramate, she continued to experience recurrent episodes of weakness and documented hypokalemia several weeks and months later, confirming that her attacks were not drug‐related.

She initially attempted oral potassium supplementation during prodromal phases, which provided partial symptomatic relief. Genetic testing (December 2024) identified heterozygosity for *CACNA1S* c.3727C  >  G (p.Leu1243Val). Detailed variant annotation is provided in Supplementary Table S1. Although initially listed as a variant of uncertain significance, her phenotype was highly consistent with HypoPP [1, 2].

She was started on spironolactone 25 mg daily and acetazolamide 250 mg four times daily in January 2025 with 40 mEq KCl twice daily and as needed. Acetazolamide reduced her episodes but caused significant side effects, including nausea, fatigue, subjective shortness of breath, and paresthesias [12]. Insurance approval was obtained for dichlorphenamide, and a transition plan was made [9].

On follow‐up, her potassium was stable at 3.8 mmol/L. Kidney function was normal (eGFR 101 mL/min/1.73 m^2^), the urine albumin‐to‐creatinine ratio was 2 mg/g, and the renal ultrasound showed normal‐sized kidneys without hydronephrosis or cysts. During one paralytic attack, she was also found to have transient hypophosphatemia, consistent with insulin‐driven intracellular shifts [13–15].

Family history was notable for electrolyte imbalances in her maternal grandfather. Family members have not undergone genetic testing; however, de novo variants are well documented in HypoPP [7, 8]. Electrodiagnostic testing, including long‐exercise CMAP or needle EMG, was discussed but declined by the patient.

She was counseled on trigger avoidance and the importance of medication adherence.

## 5. Discussion and Conclusions

This case illustrates a symptomatic HypoPP phenotype in a carrier of the rare *CACNA1S* p.Leu1243Val variant. Although most reported *CACNA1S* mutations involve S4 arginine residues, this case suggests that nearby non‐arginine substitutions can also be clinically relevant [5, 6, 16].

The *p.Leu1243Val* variant lies adjacent to the S4 arginine cluster (including p.Arg1242), a critical region for GP formation. While typical HypoPP variants produce inward GP currents during hyperpolarization, p.Arg1242Gly has been shown to generate depolarization‐activated currents associated with NormoPP [16]. Therefore, p.Leu1243Val may perturb the same structural region but result in a distinct or intermediate phenotype. Functional studies are needed to clarify its electrophysiologic impact.

Although the initial episode occurred during topiramate use, the recurrence of paralytic episodes and documented hypokalemia long after discontinuation strongly support an intrinsic channelopathy rather than secondary hypokalemia [1, 2]. The allele frequency of this variant (6.20 × 10^−7^ in gnomAD, not reported in ClinVar) further supports its rarity and potential pathogenicity [6].

Electrophysiologic confirmation via exercise testing or EMG would have been valuable, but the patient declined. Nonetheless, her clinical course, biochemical findings, and favorable response to CAIs collectively support a HypoPP diagnosis [9–11].

Reporting well‐documented cases such as these remains essential to reclassify uncertain variants and expand the known *CACNA1S* mutational spectrum [5–8].

**Nomenclature**
HypoPP: Hypokalemic periodic paralysis.
CAI: Carbonic anhydrase inhibitor.
VUS: Variant of uncertain significance.

## Consent

Written informed consent was obtained from the patient.

## Conflicts of Interest

The author declares no conflicts of interest.

## Author Contributions

Mark Abi Nader was responsible for patient care, data collection, and manuscript preparation.

## Funding

No external funding.

## Supporting Information

Table S1. Variant annotation for *CACNA1S* c.3727C > G (p.Leu1243Val), including associated phenotypes, inheritance patterns, zygosity, and classification.

## Supporting information


**Supporting Information** Additional supporting information can be found online in the Supporting Information section.

## Data Availability

All data supporting the findings of this study are included in this article and Supporting Information.
